# Supplementary material for: Ultimate limit in size and performance of WSe2 vertical diodes
Source: Nat Commun. 2018 Dec 18;9:5371. doi: 10.1038/s41467-018-07820-8 (PMC6299081; doi:10.1038/s41467-018-07820-8)
Supplement: Supplementary file 1 — Supplementary Information [file 41467_2018_7820_MOESM1_ESM.pdf]

Supplementary Information

Ultimate Limit in Size and Performance of WSe_2 Vertical Diodes

Ghazanfar Nazir[1,2], Hakseong Kim[1], Jihwan Kim[1], Kyoung Soo Kim[3], Dong Hoon Shin[4], Muhammad Farooq Khan[1,2], Dong Su Lee[3], Jun Yeon Hwang[3], Chanyong Hwang[1], Junho Suh[1], Jonghwa Eom[2] and Suyong Jung[1]

[1]Quantum Technology Institute, Korea Research Institute of Standards and Science, Daejeon 34113, Korea

[2]Department of Physics and Astronomy, Sejong University, Seoul 05006, Korea

[3]Korea Institute of Science and Technology, Jeonbuk 55324, Korea

[4]Department of Physics, Ewha Womans University, Seoul 03760, Korea

Supplementary Figures

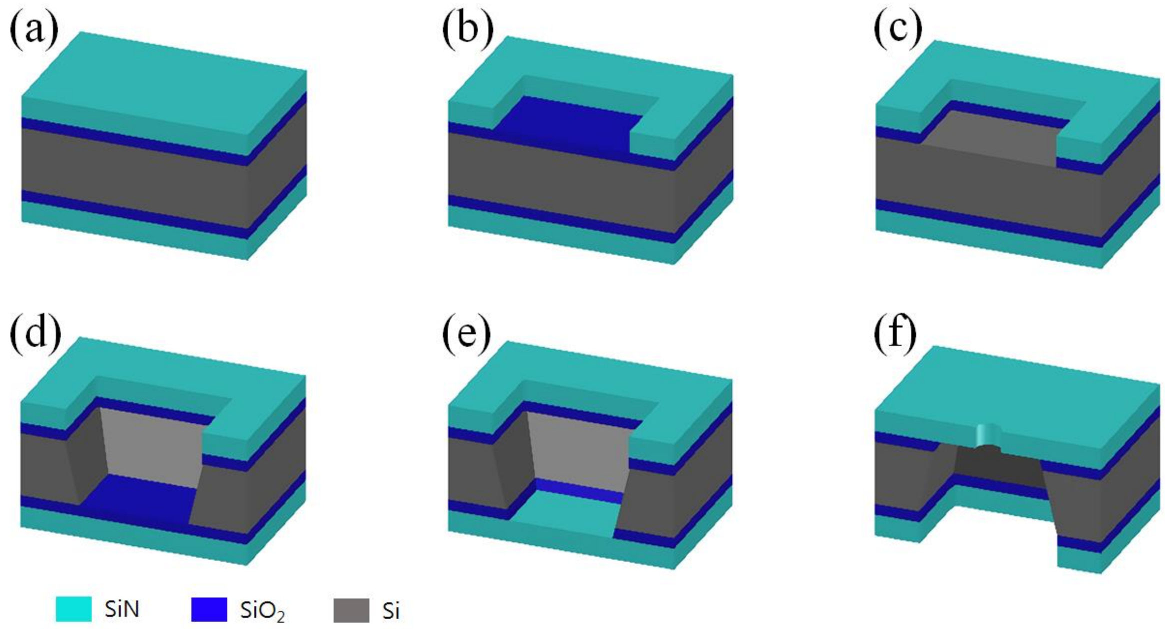

Supplementary Figure 1. Procedure for fabricating SiN through-holes. (a) A high-resistivity silicon (Si) wafer with 300 nm thick low stress SiN and 100 nm thick SiO_2 films grown on both sides of the wafer is used. (b) Photoresist (S1813) is spin-coated on the backside of the wafer and squared patterns 730 μm × 730 μm in size are defined by conventional photolithography and development process. The unmasked SiN film is etched in a reactive ion etching (RIE) system with CF_4 and Ar. (c) After removing the un-patterned photoresist with PG remover at 80 °C for 2 hours, the uncovered SiO_2 film is removed in 6:1 diluted buffered oxide etchant (BOE) for 2 minutes. (d) The 525 μm thick silicon wafer is anisotropically etched for 10 hours in 20% potassium hydroxide (KOH) aqueous solution heated to 80 ^{o}C. (e) The remaining SiO_2, which works as an etch-stop layer, is removed in 6:1 diluted BOE solution for 2 minutes. A 50 μm × 50 μm square of suspended SiN membrane is released on the Si platform. (f) A circular-shaped through-hole is patterned by electron beam lithography using a ZEP 520A resist, and the exposed SiN layer is etched with RIE. Finally, the SiN membrane with a 3 μm through-hole is completed after removing the e-beam resist in warm PG remover (80 °C) for 1 hour.

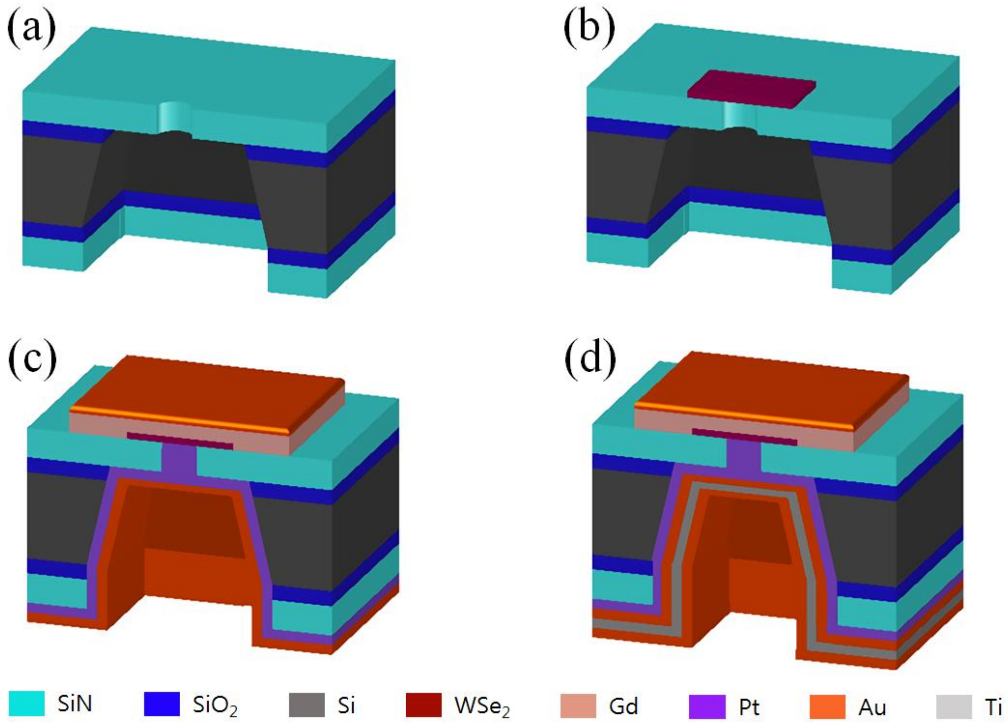

Supplementary Figure 2. Procedure for fabricating WSe_2 vertical diodes. (a) A SiN membrane (50 μm × 50 μm) with a 3 μm diameter through-hole fabricated at the center of a Si substrate (5 mm × 5 mm) is used as the experimental platform for WSe_2 vertical diodes. (b) Monolayer to multilayer WSe_2 flakes are mechanically exfoliated on stacks of PMMA (poly(methyl Methacrylate))/PSS (poly-styrene sulfonic) layers, and then transferred onto the SiN membrane by dry transfer methods. The PMMA film is removed in warm acetone for an hour and the flake is annealed at 250 ^{o}C for 7 hours in a mixture of Ar : H_2 = 9 :1 to ensure residue-free suspended WSe_2 surfaces. (c) Gd/Au and Pt/Au films are deposited on the top and the bottom surfaces of the suspended WSe_2 flake at less than a 0.3 Å/sec evaporation rate in a good vacuum (≤ 10^{-7} Torr) condition with an electron beam evaporator. (d) Bottom Si hole is filled with sputtered Ti/Au (≈ 20 nm/≈ 400 nm) films for a stable electrical connection.

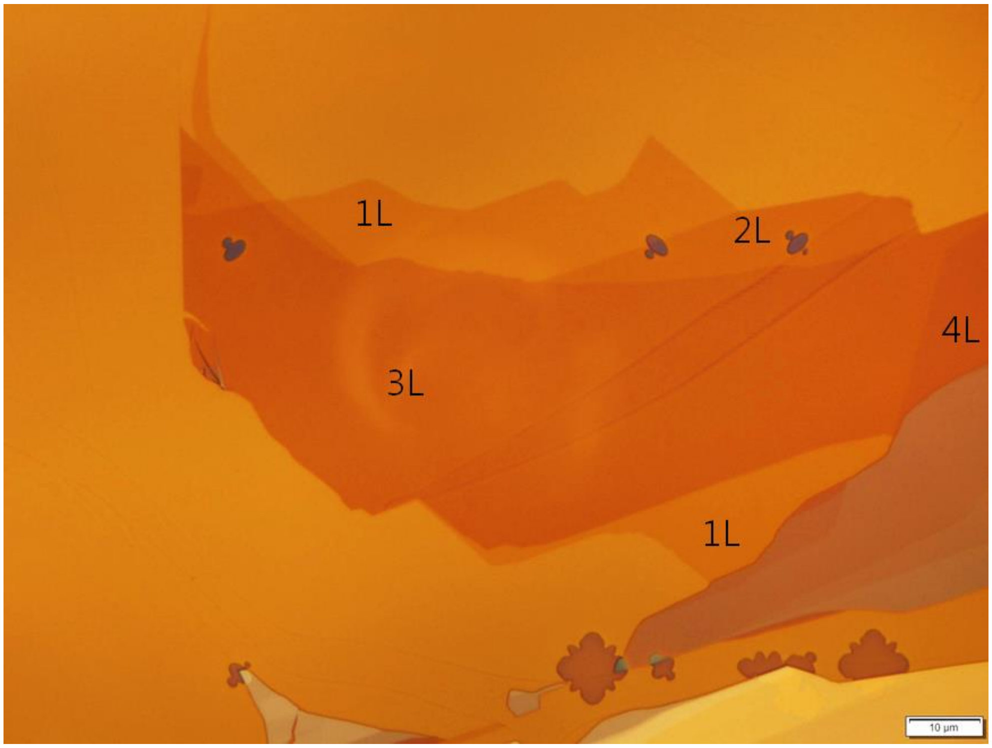

Supplementary Figure 3. Layer number identification with optical contrast. Optical microscope image of WSe_2 flakes exfoliated on PMMA/PSS polymer stacks. The scale bar is 10 μm and the layer number of WSe_2 flakes can be easily identified by optical contrasts.

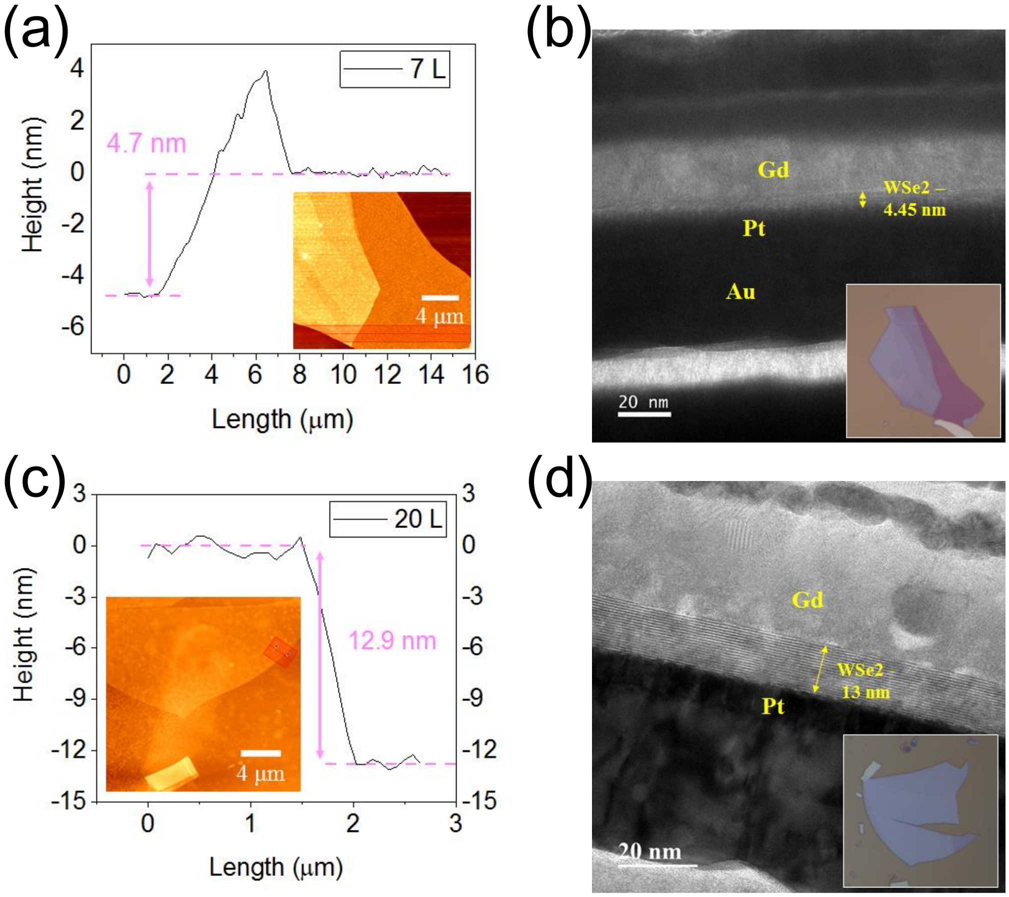

Supplementary Figure 4. Layer number identification with AFM and TEM analyses. (a, c) Supplementary Figures 4(a) and 4(c) show the AFM height profiles for 7 and 20 layers of WSe_2 flakes. (b, d) Supplementary Figures 4(b) and 4(d) display cross-sectional TEM images corresponding to the vertical devices fabricated with the identical WSe_2 flakes in the Supplementary Figures 4(a) and 4(c), respectively.

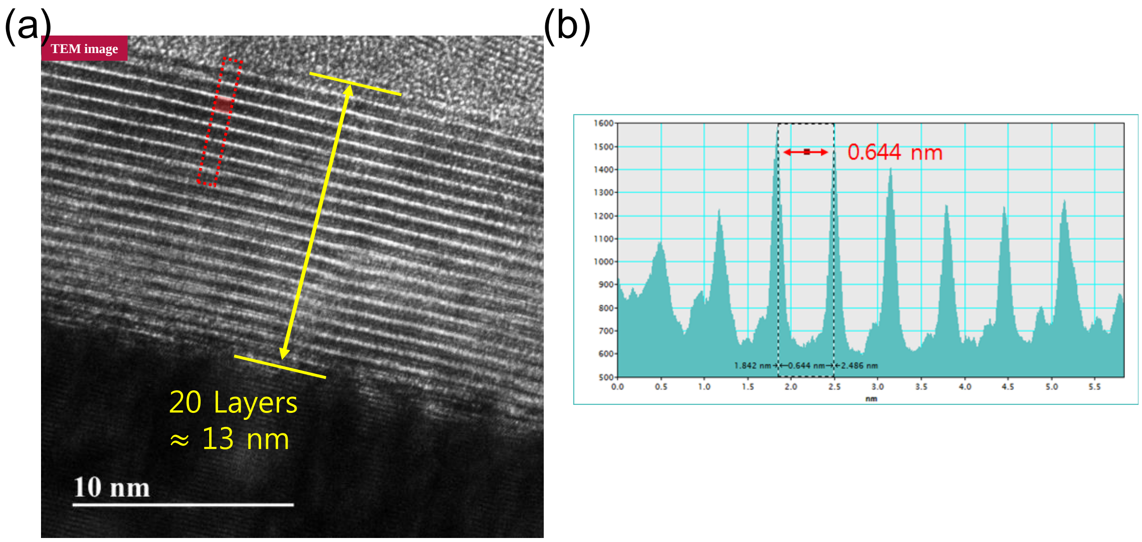

Supplementary Figure 5. HR-TEM analysis for the vertical diode with 20 layers of WSe_2. (a) High-resolution TEM (HR-TEM) image clearly revealing the layer-by-layer structure of a 13 nm thick WSe_2 flake. The yellow arrow indicates the region of WSe_2, and the upper and lower parts of the image respectively show the atomic structures of Gd and Pt films. (b) The unit thickness for monolayer WSe2 film is estimated to be around 0.644 nm.

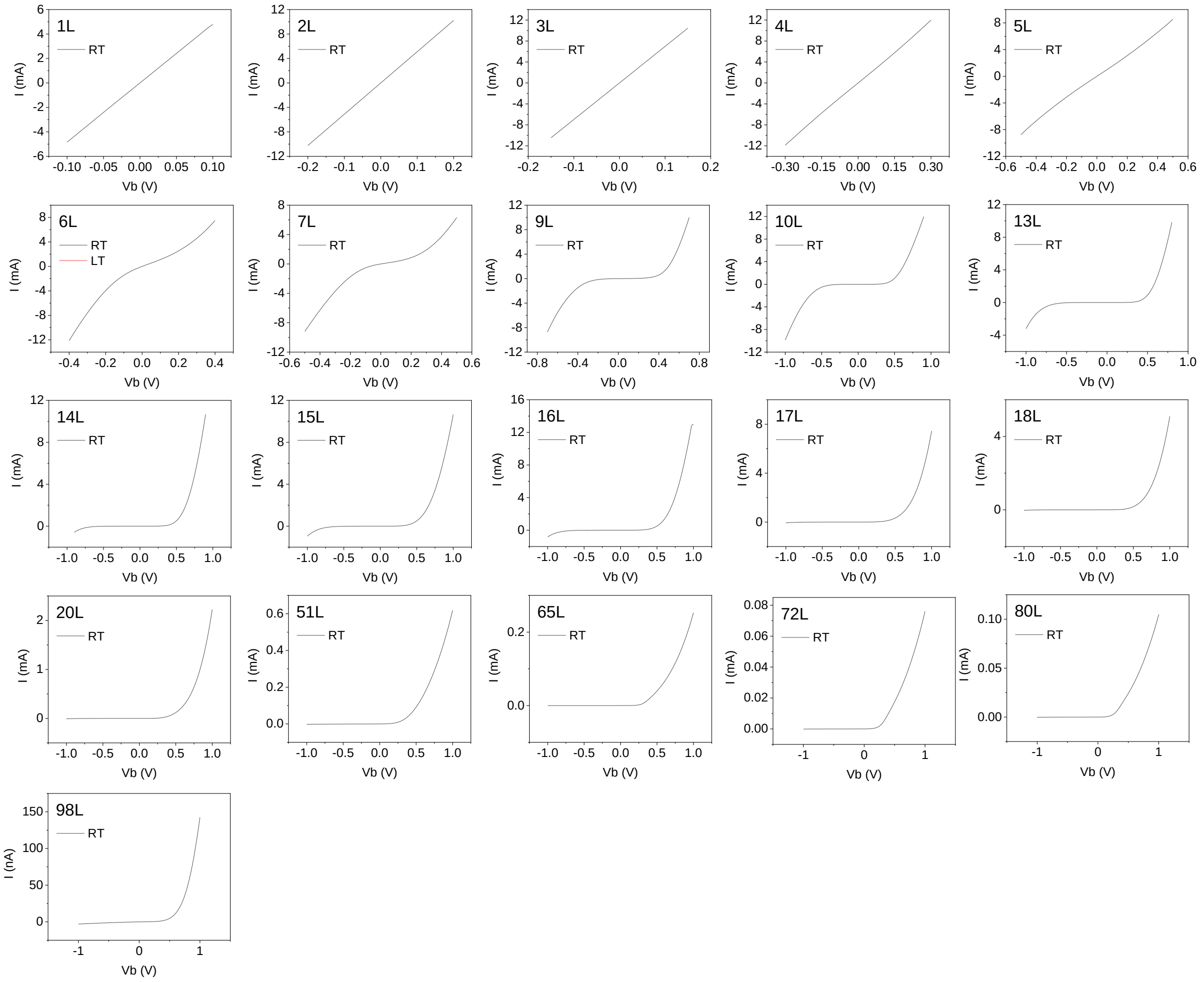

Supplementary Figure 6. *I*-V_b characteristic curves with varying layer number at room temperature. A collection of *I*-V_b characteristic curves from the WSe_2 vertical diodes with varying layer thickness at T = 300 K. Up to five layers, *I*-V_b characteristic curves show linear behaviors at T = 300 K. Junction resistances are dominated by the WSe_2–Gd and WSe_2–Pt contacts and the wire resistance of our measurement system. For the devices with 6 to 10 layers, *I*-V_b curves become nonlinear, but symmetric around V_b = 0 mV in forward and backward bias directions. As the layer number increases to more than 10L, however, reasonable diode characteristics, as represented by current rectification, are observed in the vertical junctions.

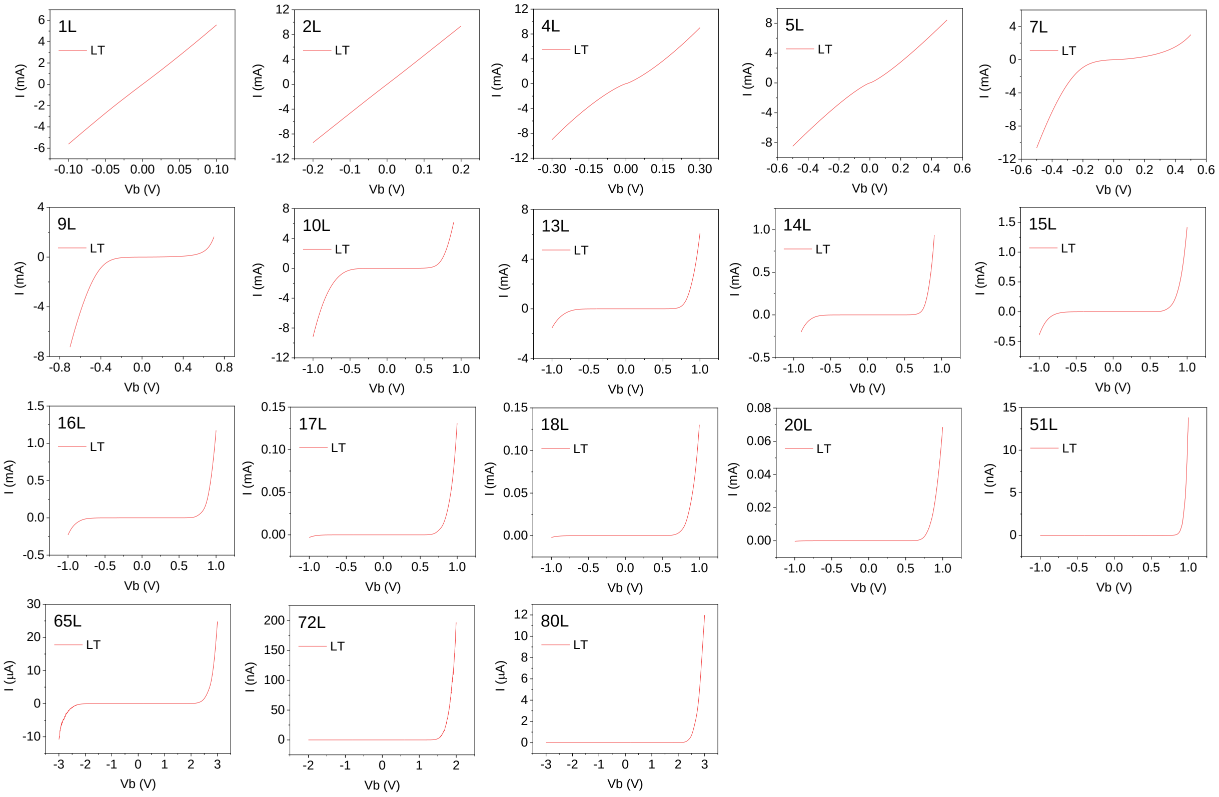

Supplementary Figure 7. I-V_b characteristic curves with varying layer number at low temperature. A collection of I-V_b characteristic curves from the WSe_2 vertical diodes with varying layer thickness at T = 6 K.

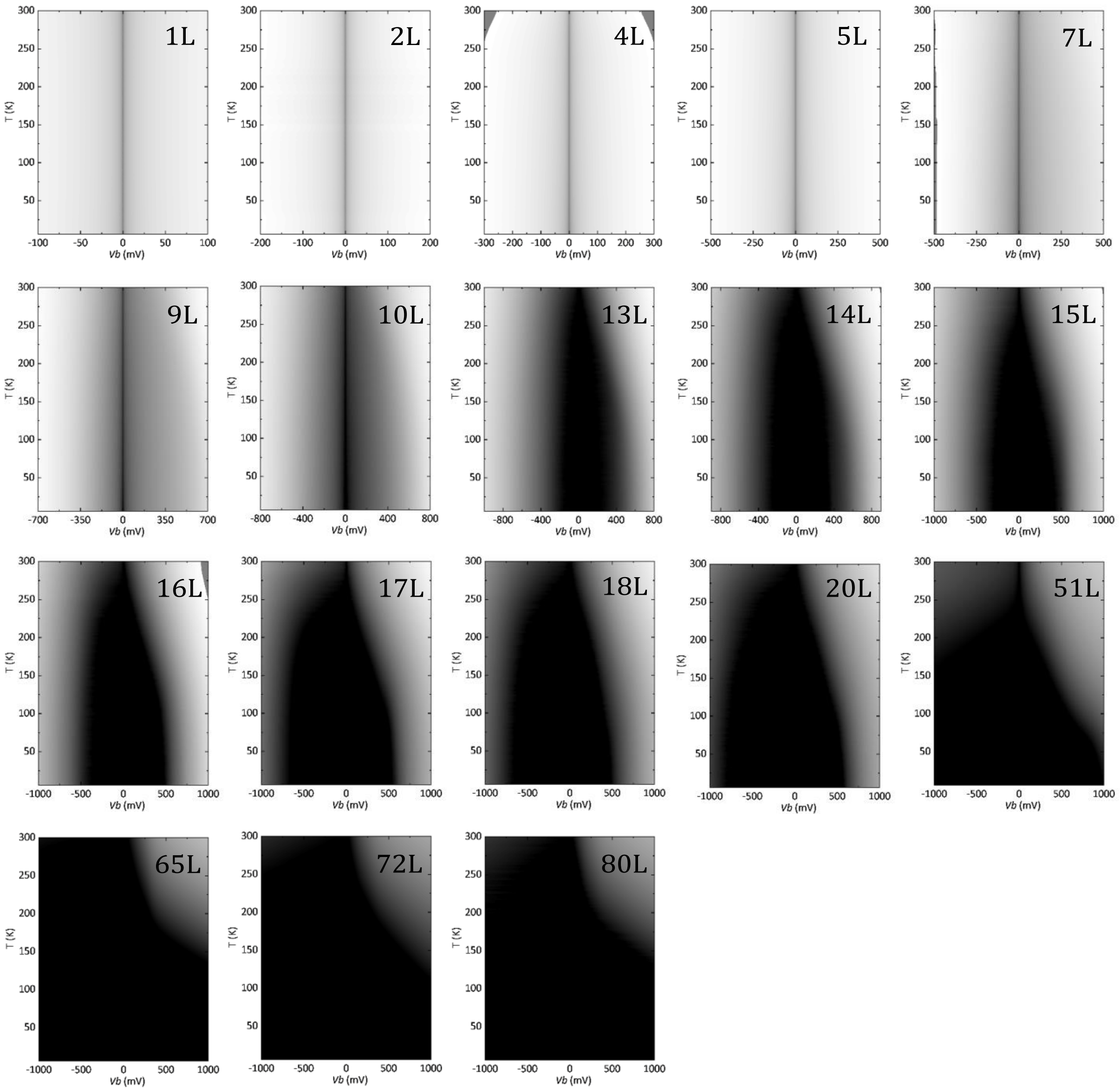

Supplementary Figure 8. Two-dimensional I-V_b mappings at various vertical diodes. A collection of two-dimensional display of I-V_b characteristic curves at increasing temperature from T = 6 K to T = 300 K with a spacing of ΔT = 2 K for WSe_2 vertical diodes of various thicknesses. Up to 9 layers, little temperature dependence is observed in the I-V_b curves, indicating that DT and FN tunneling mechanisms are dominant. When the flakes exceed 10 layers in thickness, SE tunneling starts contributing, resulting in the I-V_{SD} curves becoming temperature dependent.

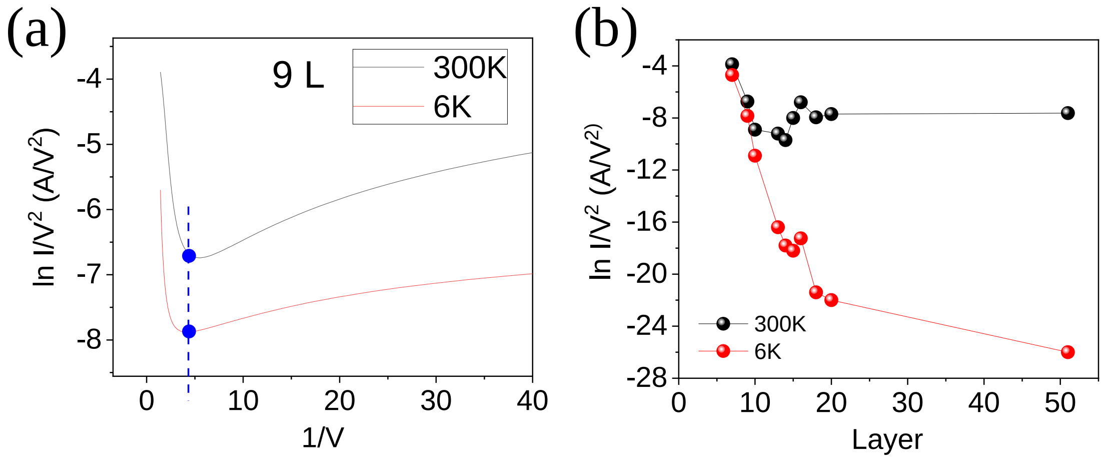

Supplementary Figure 9. FN tunneling vs. SE tunneling events. (a) FN tunneling relation plots of ln(I/V_{SD}^2) vs. 1/V_{SD} from the vertical device with a 9L flake at T = 300 K (black) and T = 6 K (red). Since vertical transport via FN tunneling is independent of temperature variation, the difference in field-emission levels at varying temperatures can be used as a direct indicator of the extent that thermally activated carriers contribute to vertical charge flows. (b) Field-emission value differences at the lowest point in ln(I/V_{SD}^2) curves at T = 6 K (marked with a dotted blue line in Supplementary Figure 9(a)) at varying layer thickness.

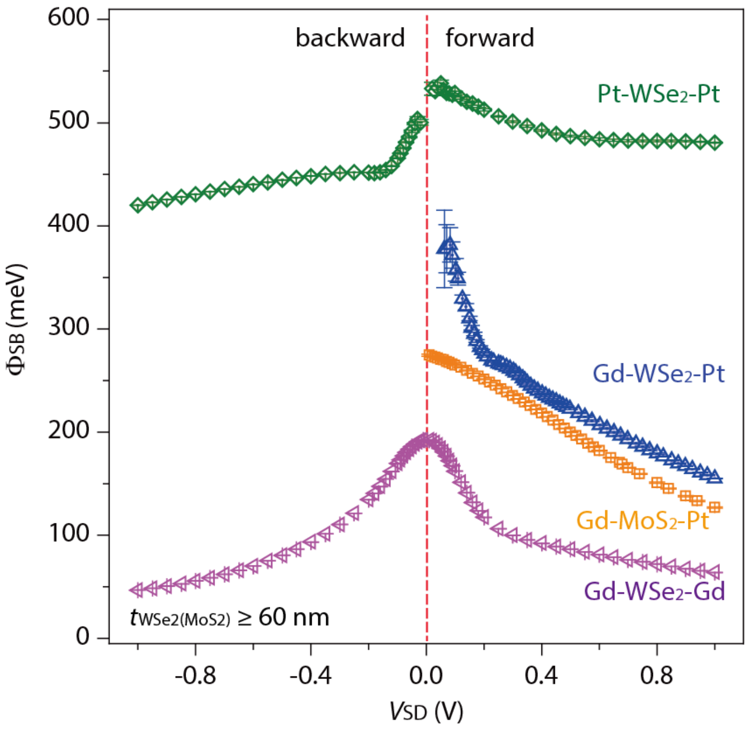

Supplementary Figure 10. Effective Schottky barrier heights. Effective Schottky barrier heights at varying V_{SD} from various vertical junctions with WSe_2 and MoS_2. SE tunneling is extremely sensitive to the height of Schottky barriers formed at the metal–semiconductor junctions. Effective Schottky barrier height (Φ_{SB}) can be extracted from the relation of current versus temperature at a fixed source-drain voltage.

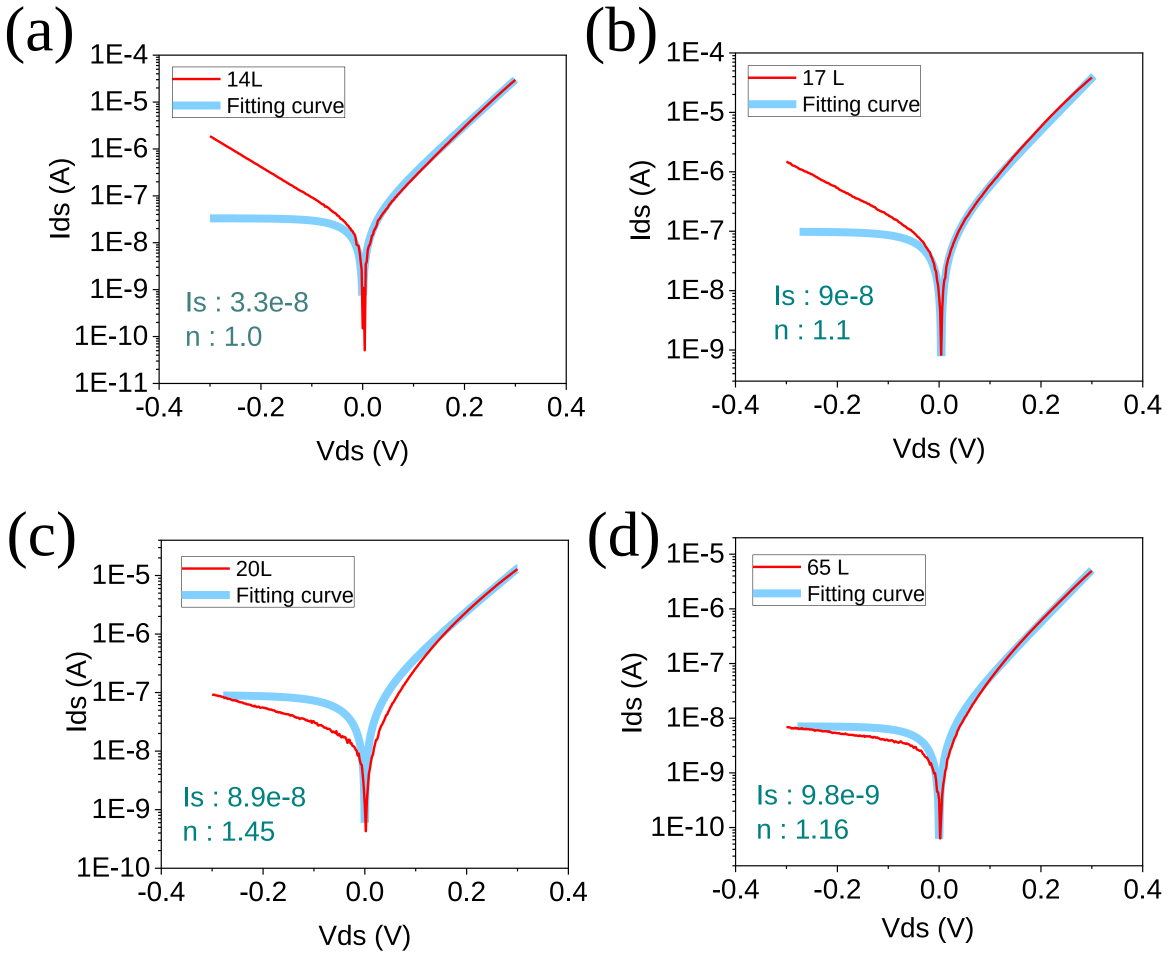

Supplementary Figure 11. I-V_{SD} characteristic curves and fittings to the Shockley diode relation from the vertical junctions of (a) 14L, (b) 17L, (c) 20L and (d) 65L of WSe_2.

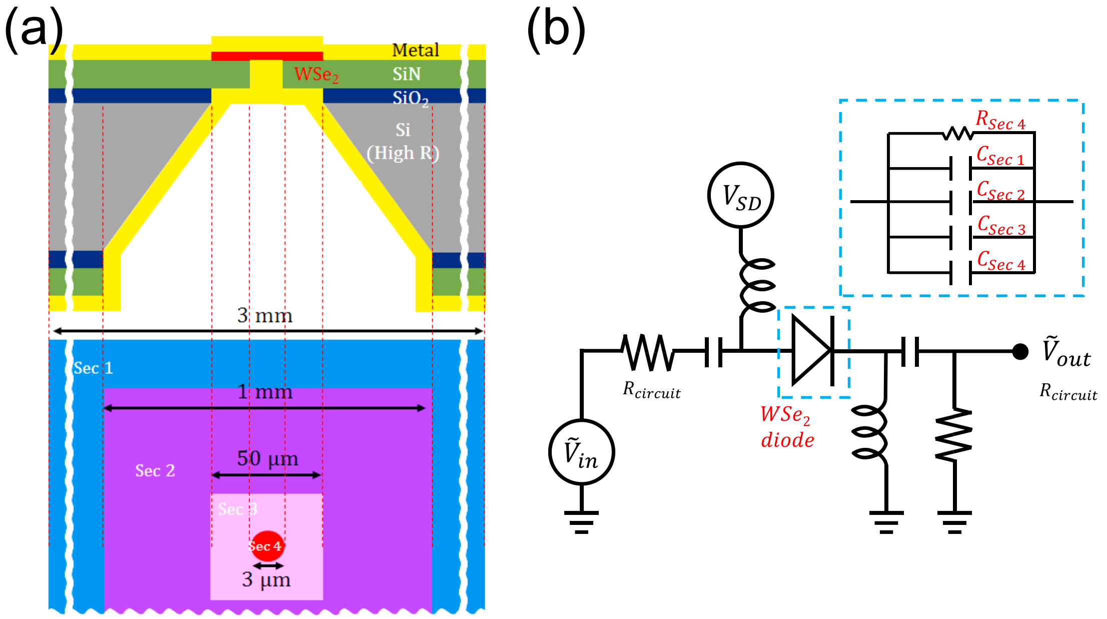

Supplementary Figure 12. Radio-Frequency (RF) switch operations with vertical WSe_2 diodes. (a) Schematics of the WSe_2 vertical diode fabricated on SiN/SiO_2 grown on high resistivity silicon substrate. (b) Schematic diagram of the electrical circuit for RF measurements.

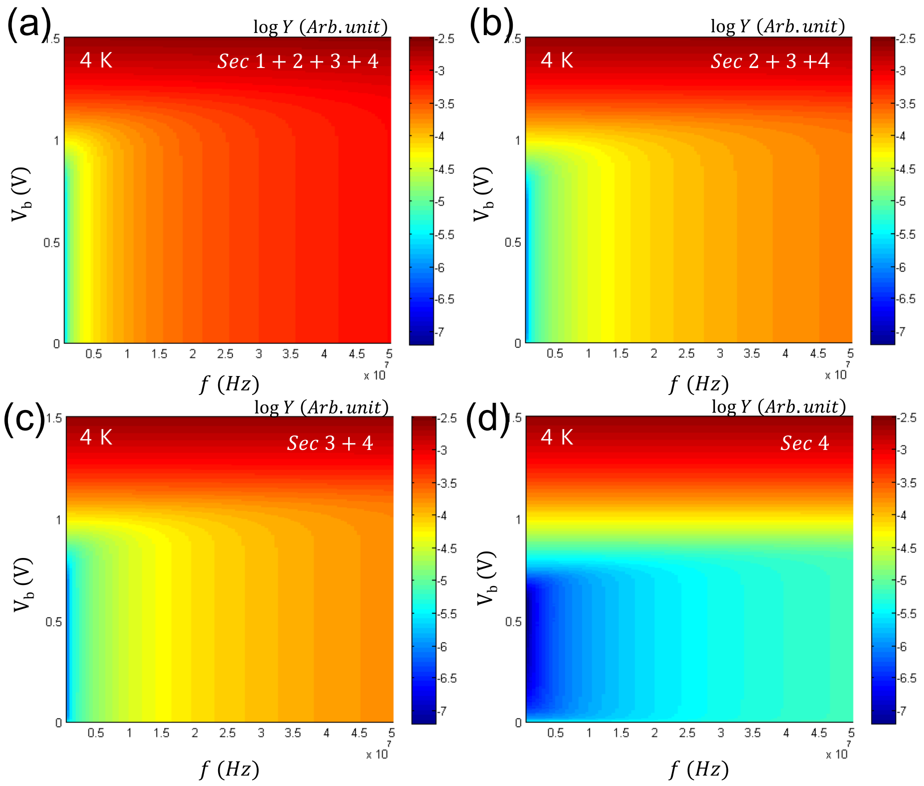

Supplementary Figure 13. Simulation results of AC admittance variations up to $f \leq 50$ MHz at T = 4 K expected from the 12 nm thick WSe_2 diode considering different junction capacitances.

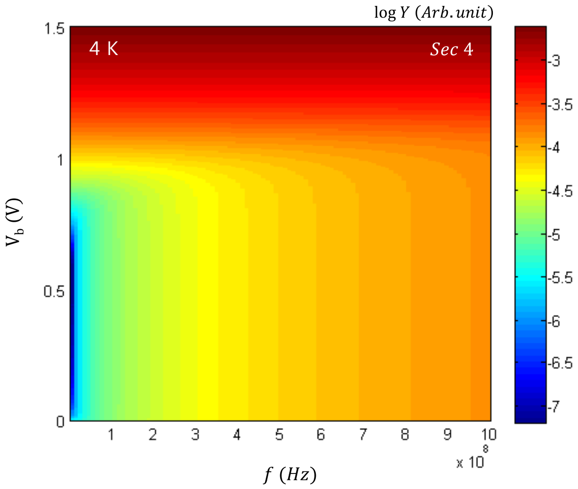

Supplementary Figure 14. Simulation result of AC admittance variations up to $f \leq 1$ GHz at T = 4 K considering only the capacitive coupling through the active WSe_2 area (junction area = 7.068 μm^2).

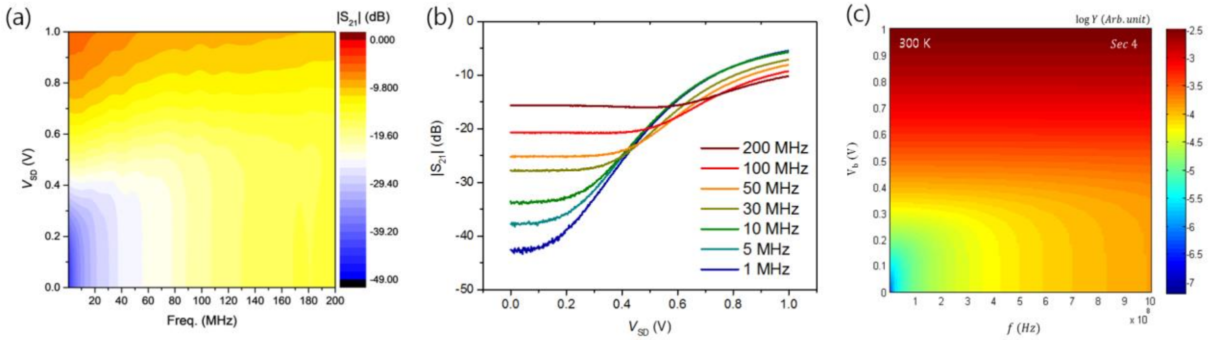

Supplementary Figure 15. (a) Transmission coefficient responses to a forward V_{SD} by varying frequencies at T = 300 K, where the RF switching property is observed up to f = 200 MHz. The transmission coefficient $|S_{21}|$ varies at different frequency regimes. (b) At f = 1 MHz, -43 dB of isolation and a -5 dB of insertion loss can be characterized for the OFF and ON state of the switching operation. However, the difference between the ON and OFF states gradually reduces with increasing frequencies. (c) Simulation result of AC admittance variations up to $f \leq 1$ GHz at T = 300 K considering only the capacitive coupling through the active WSe_2 area (junction area = 7.068 μm^2).

Supplementary Table

	Dielectric constant
ε_{Nit}	7.5
ε_{SiO2}	3.9
ε_{Si}	11.8
ε_{WSe2}	4

	Thickness (m)
WSe_2	12×10^{-9}
SiN	300×10^{-9}
SiO_2	100×10^{-9}
Si	525×10^{-6}

	Area (m^2)
$Sec\ 1$	8×10^{-6}
$Sec\ 2$	9.98×10^{-7}
$Sec\ 3$	2.49×10^{-9}
$Sec\ 4$	7.07×10^{-12}

	Capacitance (F)
$Sec\ 1$	1.57×10^{-12}
$Sec\ 2$	9.92×10^{-14}
$Sec\ 3$	5.13×10^{-13}
$Sec\ 4$	2.1×10^{-14}

Supplementary Table 1. Materials and structural information used for simulating RF switching operations in our WSe_2 vertical diodes

Supplementary Notes

Supplementary Note 1: Characterizing WSe_2 vertical didoes

We characterize our WSe_2 vertical diode operations with the Schokley diode model as described below

$$I = I_{sat}\left[exp\left(\frac{qV}{nk_BT}\right) - 1\right], \qquad \text{(Supplementary Equation 1)}$$

where I_{sat} is the reverse-bias saturation current, and n is the ideality factor. Since two-probe diode operations are easily limited by the contact resistance in metal and semiconductor junctions, preparation of an atomically clean metal–semiconductor junction is important to realize an ideal diode, especially for the cases of 2D semiconductors. In our vertical junctions, residue-free interfacial regions are achieved through direct metal evaporations onto homogeneous WSe_2 flakes. Thus, the ideality factor n estimated in the bias voltage range of $V_{SD} \leq 0.3$ V is close to the ideal case of $n \approx 1.0$ for most of the devices, with little variations depending on the vertical junction thickness, as shown in the fitting data in Supplementary Figure 11.

Supplementary Note 2: Characterizing RF switching operations

As a fundamental diode application, RF switching operations of the WSe_2 vertical diode 12 nm in thickness are investigated at a cryogenic temperature of $T = 4.2$ K. As described in the main text, our vertical diodes reveal reasonable switching operations up to $f \leq 300$ MHz with a threshold voltage at $V_{SD} \approx 0.8$ V, with the primary limiting factor in high-frequency operations being signal loss through the Si/SiN substrate. As illustrated in Supplementary Figure 12, RF operations in our vertical junctions are determined by the junction resistance and capacitance (C_{sec4}), which are in turn determined by the WSe_2 flake itself and the parallel connected capacitances through the silicon nitride membrane (C_{sec3}), etched Si substrate (C_{sec2}), and un-etched (C_{sec1}) Si substrate. To investigate the contributions of each component to the RF-switching operations, we establish a simple analytical model of a parallel-connected RC circuit (Supplementary Figure 12(b)). We consider that the resistances of SiN, SiO_2, and Si are infinite,

which is especially true for high-resistivity Si wafers at cryogenic temperatures. Therefore, the total capacitance and resistance of the vertical diodes can be described as

$$C_{\text{tot}} = C_{\text{Sec1}} + C_{\text{Sec2}} + C_{\text{Sec3}} + C_{\text{Sec4}} \quad \text{(Supplementary Equation 2)}$$

$$R_{\text{tot}} = R_{\text{WSe2}}, \quad \text{(Supplementary Equation 3)}$$

and the capacitance for each section is calculated as follows,

$$\frac{1}{C_{\text{Sec1}}} = \frac{2}{C_{\text{SiN}}} + \frac{2}{C_{\text{SiO2}}} + \frac{1}{C_{\text{Si}}}$$

$$\frac{1}{C_{\text{Sec2}}} = \frac{1}{C_{\text{SiN}}} + \frac{1}{C_{\text{SiO2}}} + \frac{1}{2C_{\text{Si}}}$$

$$\frac{1}{C_{\text{Sec3}}} = \frac{1}{C_{\text{SiN}}} + \frac{1}{C_{\text{WSe2}}}$$

$$\frac{1}{C_{\text{Sec4}}} = \frac{1}{C_{\text{WSe2}}}. \quad \text{(Supplementary Equation 4)}$$

With estimated capacitance values, we calculate the admittance of the junction by following Supplementary Equation 5; two-dimensional contour plots of AC admittance in our vertical diodes are expressed as functions of DC bias voltage and AC frequency. In these simulations, we use the same R_{tot} obtained from the I-V_{SD} curves of the 12 nm thick WSe_2 vertical junction at T = 4.2 K.

$$Y_{\text{tot}} = \frac{1}{\sqrt{\left(\frac{1}{\frac{1}{R_{\text{tot}}} + R_{\text{tot}}\omega^2 C_{\text{tot}}^2}\right)^2 + \left(\frac{\omega C_{\text{tot}}}{\left(\frac{1}{R_{\text{tot}}}\right)^2 + \omega^2 C_{\text{tot}}^2}\right)^2}} \quad \text{(Supplementary Equation 5)}$$

As shown in Supplementary Figure 13(a), the simulation result of AC admittance looks quite similar to our experimental data with a cut-off frequency at $f \leq 300$ MHz and a threshold voltage at $V_{\text{SD}} \approx 0.8$ V when we consider all existing capacitances: $C_{\text{tot}} = C_{\text{sec1}} + C_{\text{sec2}} + C_{\text{sec3}} + C_{\text{sec4}}$. As sequentially displayed in the AC-admittance plots of Supplementary Figures 13(b)–13(d), the cut-off frequencies expected from our vertical diodes gradually increase, and the on/off switching operations improve as the junction capacitances are eliminated one by one.
